# Treatment Sequencing in ARPI-Pre-Exposed Metastatic Castration-Resistant Prostate Cancer: Radioligand Therapy, PARP Inhibitors, and Antibody–Drug Conjugates

**DOI:** 10.3390/cancers18193119

**Published:** 2026-09-25

**Authors:** Baha Sharaf, Mohammad Al-Rwashdeh, Osama El Khatib, Akram Al-Ibraheem, Ala Abu Fara, Ameen Ismail, Hadeel Alkasrawi, Mohamed Abdalla, Sharif Jehad

**Affiliations:** 1 Department of Internal Medicine, King Hussein Cancer Center (KHCC), Amman 11941, Jordan; 2 Department of Nuclear Medicine, King Hussein Cancer Center (KHCC), Amman 11941, Jordan; 3 Faculty of Medicine, Yarmouk University, Irbid 21163, Jordan

**Keywords:** prostate cancer, treatment sequencing, PSMA radioligand therapy, lutetium-177, PARP inhibitors, antibody–drug conjugates, BRCA2, neuroendocrine prostate cancer, circulating tumour DNA

## Abstract

**Simple Summary:**

Men whose prostate cancer stops responding to hormone-blocking treatment now have several further options, including targeted tablets for those with inherited or tumour DNA-repair faults, a radioactive drug that seeks out a protein on prostate cancer cells, and chemotherapy. No trial has compared these options head to head in the order they should be given. Almost all patients now receive a potent hormone-blocking drug earlier in their illness, which means the evidence guiding these later choices was generated in patients who differ from those seen today. This review sets out what the trials do and do not show and proposes a provisional framework for discussion between clinicians and patients. It is not a treatment guideline.

**Abstract:**

**Background/Objectives:** Androgen receptor pathway inhibitor (ARPI) intensification in metastatic hormone-sensitive prostate cancer has changed the population reaching metastatic castration-resistant prostate cancer (mCRPC), and the optimal sequence of subsequent therapies is uncertain. **Methods:** The study presents a narrative review of phase II/III trials reporting radiographic progression-free survival (rPFS) or overall survival (OS) in mCRPC, including congress data, to June 2026, graded on a five-level evidence hierarchy. **Results:** No prospective sequencing trial has been reported. CARD (cabazitaxel versus an ARPI switch after docetaxel; OS HR 0.64) remains the clearest sequencing dataset. Alpha-controlled OS benefit for PARP inhibitors is established in the PROfound cohort A (HR 0.69) and in the TALAPRO-2 all-comer population (HR 0.80); larger BRCA-specific estimates are exploratory, and BRCA OS in TRITON3 was not improved (HR 0.91). ^177^Lu-PSMA-617 improved rPFS versus an ARPI switch in PSMAfore, but intention-to-treat OS did not differ (HR 0.91) amid 60% crossover; no taxane-controlled trial has shown an OS advantage, and in preliminary PLUDO data, OS favoured docetaxel first (HR 1.64), a difference potentially confounded by asymmetric crossover. **Conclusions:** We propose a hypothesis-generating four-track framework stratified by homologous recombination repair status and PSMA-PET expression; treatment choice remains individualised.

## 1. Introduction

Managing mCRPC in 2026 is harder than it was a decade ago, not because the options are poor but because there are now more of them than the available evidence knows how to order.

Approved agents include abiraterone, enzalutamide, docetaxel, cabazitaxel, radium-223, olaparib, rucaparib, niraparib, talazoparib and ^177^Lu-PSMA-617. Several, though not all, have demonstrated an overall survival benefit in the specific mCRPC populations in which they were tested; others were approved on radiographic progression-free survival, and the survival evidence differs by agent and by biomarker subgroup, as set out in Section 3, Section 4 and Section 5 [1,2]. The question no trial has yet answered is in what order to use them.

Between 2015 and 2024, phase III trials established that adding an ARPI to androgen-deprivation therapy (ADT) extends survival in mHSPC [3,4,5,6,7,8]. CHAARTED established this for docetaxel and LATITUDE for abiraterone, and ARCHES, TITAN, ARASENS and PEACE-1 extended it to enzalutamide, apalutamide, darolutamide and triplet combinations. By 2024, ADT plus an ARPI had become the minimum standard for most patients with mHSPC.

That shift reshaped the mCRPC population. Most patients who progress to castration resistance have already received abiraterone or enzalutamide, and the evidence that a second ARPI adds little in this setting is reasonably consistent, though not uniform. In the randomised phase II crossover trial of Khalaf and colleagues, PSA responses to second-line ARPI therapy were seen in 36% of patients receiving enzalutamide after abiraterone but in only 4% of those receiving abiraterone after enzalutamide [9]. The asymmetry matters: the enzalutamide-after-abiraterone sequence retained meaningful, if modest, activity, and the case against ARPI-to-ARPI sequencing is therefore stronger in one direction than the other. The PLATO trial, which tested whether continuing enzalutamide alongside abiraterone could overcome enzalutamide resistance, found no benefit (rPFS HR 0.83, 95% CI 0.61–1.12; *p* = 0.22) and concluded against the combination [10]. The 2025 EAU guidelines advise against sequencing androgen-receptor-targeted agents, as a weak recommendation [11]. The realistic post-ARPI options are therefore PARPi for HRR-mutated disease, ^177^Lu-PSMA-617 for PSMA-positive disease, and taxane chemotherapy.

Three developments have sharpened the sequencing problem further. PSMAddition showed that ^177^Lu-PSMA-617 extends rPFS even in the hormone-sensitive setting [12], so RLT may migrate upstream and compress the options available at the mCRPC transition. The randomised PR.21 trial (PLUDO) provided the first direct comparison of RLT against docetaxel in mCRPC [13]. And ABBV-969, a bispecific PSMA × STEAP1 ADC, produced a preliminary objective response rate (ORR) of 45% in a heavily pre-treated population that included patients previously treated with RLT [14].

This review synthesises the available mature OS evidence, integrates 2025–2026 congress data with explicit labelling of their limitations, and proposes a hypothesis-generating framework. It also defines the prospective trials the field still requires.

### 1.1. Scope and Methodology

This is a narrative review. PRISMA methodology was not applied, and no formal risk-of-bias assessment was performed. Both would have increased methodological rigour; neither was feasible within the timeframe required to incorporate 2026 congress data. The search strategy and selection rules are therefore reported in full so that readers can appraise the choices made.

We searched PubMed, EMBASE and ClinicalTrials.gov, and reviewed oral presentations and abstracts from the ASCO, ASCO GU, ESMO and EAU meetings from January 2022 to June 2026. Before submission, the search was updated in September 2026 for full publications of trials already identified, which led to incorporation of the peer-reviewed PSMAddition report. Search terms comprised metastatic castration-resistant prostate cancer, treatment sequencing, PARP inhibitor, radioligand therapy, lutetium PSMA, antibody–drug conjugate, cabazitaxel, HRR, BRCA2, neuroendocrine prostate cancer, AR-V7, ctDNA, CDK12, and the names of individual trials (PSMAfore, CARD, VISION and others).

Inclusion criteria were (i) phase II or III randomised controlled trials reporting rPFS or OS in mCRPC or mHSPC; (ii) single-arm phase I or phase II studies with mature response data judged informative for sequencing decisions in the populations enrolled; (iii) biomarker or ctDNA studies of direct clinical applicability; and (iv) EAU, ASCO or ESMO guideline updates from 2022 to 2026. Exclusion criteria were single-arm studies with fewer than 20 evaluable patients, unless uniquely relevant to an underserved subgroup; preclinical-only studies; expert opinion without primary data; and case reports.

Negative trials were not excluded. The limited and uncertain evidence for PARPi monotherapy benefit in CDK12-altered disease, the futility of niraparib in the non-BRCA HRR cohort of MAGNITUDE, the non-significant intention-to-treat OS result of PSMAfore, the null final OS in the BRCA subgroup of TRITON3 and the non-significant primary rPFS endpoint of PLUDO are all reported in full. Final trial selection was made by consensus among three authors (B.S., M.A.-R., A.A.-I.), with disagreements resolved by discussion. No independent review panel was used, which is a limitation.

### 1.2. Evidence Hierarchy

All clinical statements in this review carry one of the five evidence labels defined in Table 1. The hierarchy is applied uniformly: where a result derives from a post hoc, exploratory or unstratified subgroup analysis, it is graded [Low] regardless of the phase of the parent trial and regardless of the size of the effect estimate. This rule is applied to the BRCA subgroup results of PROfound, PROpel and TALAPRO-2, each of which is an exploratory subgroup analysis rather than an alpha-controlled endpoint.

Evidence labels describe the strength and applicability of the evidence supporting each specific clinical statement; they do not, by themselves, establish that a test or intervention is standard of care. Guideline-supported assessments and investigational approaches are distinguished separately throughout the framework.

### 1.3. Limitations of Congress-Derived Evidence

A substantial part of the most recent evidence discussed here exists only in congress form and has not been peer reviewed. This includes ABBV-969, AcTION, the PLUDO primary and crossover analyses, the BRCAAway OS update, the ENZA-p OS analysis, the TRITON3 final OS analysis, the DB-1311 phase I/II data, the interim OS and subgroup analyses of PSMAddition, the mevrometostat randomised expansion, the LuPARP phase I data, the ARV-110 phase I/II data, and the SPLASH and ECLIPSE results. All are labelled [Preliminary]. Every numerical estimate drawn from these sources should be treated as provisional, since effect sizes and denominators reported in abstracts are frequently revised at full publication. The review does not treat congress-only results as definitive evidence, and no recommendation in Section 9 rests on congress data alone.

## 2. The Changed Patient: mCRPC After ARPI Intensification

### 2.1. Why the Prior Sequencing Literature No Longer Applies

The pivotal PARPi monotherapy trials, PROfound (2020) and TRITON3 (2023), established benefit in selected patients with previously ARPI-treated mCRPC [15,16]. Their applicability specifically after ARPI exposure in the hormone-sensitive setting requires separate consideration, as does that of the first-line combination trials discussed in Section 3.

Several AR-pathway mechanisms explain why rechallenge with an ARPI after ARPI failure frequently fails. AR-V7 is a splice variant that retains the N-terminal and DNA-binding domains but lacks the ligand-binding domain targeted by enzalutamide; it therefore renders the receptor constitutively active without a ligand [17]. Abiraterone acts by a different mechanism, inhibiting androgen synthesis through CYP17 rather than binding the receptor, and AR-V7 confers resistance to both agents because neither removes ligand-independent receptor activity. Amplification of the AR gene at Xq12 elevates receptor protein above the level that castrate androgen concentrations can suppress [18], and amplification of the upstream AR enhancer produces the same effect. The F876L/F877L point mutation converts enzalutamide from antagonist to partial agonist, so that the drug actively drives AR signalling [19]. T878A acts through a different mechanism: it broadens ligand promiscuity, permitting receptor activation by alternative ligands including progesterone, and is characteristically selected under androgen-synthesis inhibition rather than under receptor blockade [20]. The two mutations therefore arise under different selective pressures, which is relevant when choosing the next agent; the L702H mutation permits AR activation by glucocorticoids, which is clinically relevant in patients receiving prednisone with abiraterone. Any of these may emerge during ARPI exposure in mHSPC and may be present, at varying subclonal fractions, when mCRPC is declared; their prevalence at that point has not been systematically characterised in contemporary ARPI-pre-exposed cohorts.

Treatment options for mCRPC include androgen receptor–directed therapies, taxane chemotherapy and biomarker-selected treatments, including PARPi, PSMA-targeted radioligand therapy and immune checkpoint inhibition, alongside investigational approaches [21]. This list describes what is available. It does not resolve which agent should be used first or in whom (Figure 1).

### 2.2. HRR Gene Alterations Are Not Clinically Interchangeable

Approximately 20–25% of patients with mCRPC carry a pathogenic germline or somatic HRR alteration [22,23]. The gene-level frequencies cited below are drawn from different cohorts using different sequencing panels and definitions of pathogenicity, so they overlap and do not sum to the aggregate figure; they should be read as approximate rather than additive. The PSA50 response ranges quoted are likewise drawn from heterogeneous series and should be regarded as indicative rather than precise [Low].

BRCA2 (approximately 13% of mCRPC) carries the strongest and most consistent PARPi signal, and the exploratory subgroup analyses of the combination trials show the largest effect estimates in this subgroup (Section 3). Point mutations can give rise to secondary reversion mutations that restore homologous recombination function, whereas homozygous deletions cannot [24]; patients with deletions may therefore have more durable responses, though this has not been tested prospectively [Mechanistic inference].

BRCA1 (approximately 1–2%) is PARPi-sensitive, but the magnitude of response appears lower than for BRCA2. In the PROfound BRCA subgroup analysis, median rPFS with olaparib was 10.8 months in BRCA2-altered disease (*n* = 128) but 2.1 months in BRCA1-altered disease (*n* = 13), a difference based on very small numbers [25] [Low]. BRCA1 and BRCA2 should not be pooled when counselling patients.

ATM (approximately 7%) shows a substantially weaker signal, with shorter response duration and no demonstrated OS benefit. In TRITON3, ATM-altered patients derived no rPFS benefit from rucaparib (HR 0.95, 95% CI 0.59–1.52), and OS numerically favoured the control arm [16]. The mechanistic explanation is that ATM functions in early double-strand-break signalling rather than in the core homologous recombination machinery [26]; ATM-deficient prostate cancer models show variable PARPi sensitivity and greater dependence on ATR [26]. Expectations derived from BRCA2 do not transfer.

CDK12 biallelic loss occurs in approximately 5–7% of cases; evidence for PARPi monotherapy benefit in this subgroup remains limited and uncertain. This point requires careful sourcing, because TRITON3 did not enrol CDK12-altered patients: its eligibility criteria were restricted to deleterious BRCA1, BRCA2 or ATM alterations [16]. The evidence comes instead from the phase II TRITON2 non-BRCA analysis, in which no confirmed radiographic responses to rucaparib were observed among CDK12-altered patients [27], and from the gene-level analysis of PROfound, which does not establish a definitive CDK12-specific treatment effect [15]. Both analyses are limited by small subgroup sizes, so the appropriate label is [Low]. These monotherapy findings should not necessarily be generalised to PARPi–ARPI combinations, which have not been evaluated specifically in CDK12-altered disease after prior ARPI; current ASCO recommendations for PARPi are anchored in BRCA1/2 alterations [28]. CDK12 loss is associated with focal tandem duplications and increased neoantigen burden [29], and T-cell-engaging bispecifics and immunotherapy combinations are biologically more rational in this subgroup [Mechanistic inference].

CHEK2, PALB2, RAD51D, FANCA and BRIP1 (collectively approximately 3–5%) have limited prospective data. PALB2 behaves most like BRCA2; monoallelic CHEK2 confers substantially less PARPi sensitivity. Enrolment in biomarker-enriched trials is the appropriate disposition.

## 3. PARP Inhibitors: Mature Survival Data and the ARPI Pre-Exposure Problem

### 3.1. Monotherapy: PROfound and TRITON3

PROfound randomised patients with HRR-mutated, ARPI-treated mCRPC to olaparib or physician’s choice of a second ARPI [15]. In cohort A (BRCA1, BRCA2 or ATM alterations) the rPFS HR was 0.34 (95% CI 0.25–0.47). The alpha-controlled final OS analysis in cohort A showed a median OS of 19.1 versus 14.7 months (HR 0.69, 95% CI 0.50–0.97; *p* = 0.02), despite 67% crossover from control to olaparib within that cohort; a crossover-adjusted sensitivity analysis gave an HR of 0.42 (95% CI 0.19–0.91) [30] [High].

A separate post hoc exploratory analysis restricted to the 160 patients with BRCA1 or BRCA2 alterations reported an rPFS of 9.8 versus 3.0 months (HR 0.22, 95% CI 0.15–0.32) and an OS of 20.1 versus 14.4 months (HR 0.63, 95% CI 0.42–0.95) [25]. Because this analysis was post hoc and not alpha-controlled, it is graded [Low] despite arising from a phase III trial. The distinction matters: cohort A OS (HR 0.69) is the formally tested result, whereas the BRCA estimate (HR 0.63) is hypothesis-generating.

TRITON3 showed an rPFS HR of 0.50 (95% CI 0.36–0.69) in the BRCA1/2 subgroup, with a median rPFS of 11.2 versus 6.4 months, and an ITT rPFS HR of 0.61 (95% CI 0.47–0.80) [16] [Moderate]. In the final OS analysis presented at ASCO GU 2025, OS in the BRCA subgroup was similar between arms (23.2 versus 21.2 months; HR 0.91, 95% CI 0.68–1.20), and in the ATM subgroup rPFS was not improved (HR 0.95, 95% CI 0.59–1.52), with OS numerically favouring the control arm (HR 1.21, 95% CI 0.77–1.90) [31] [Preliminary]. The investigators attributed the absence of an OS difference partly to extensive crossover from the control arm to rucaparib.

The null BRCA OS result in TRITON3 is an important counterweight to the PROfound and PROpel subgroup estimates and is the principal reason this review does not describe the BRCA OS evidence as uniformly large and consistent.

### 3.2. First-Line Combinations: Mature Overall Survival Is Now Available

The major first-line PARPi plus ARPI combination trials now have mature OS data, but the magnitude and consistency of benefit differ by biomarker and trial, and the biomarker-defined estimates are exploratory.

PROpel (olaparib plus abiraterone) reported an ITT OS of 42.1 versus 34.7 months (HR 0.81, 95% CI 0.67–1.00, *p* = 0.054), which did not meet the prespecified significance threshold in the all-comer population [32] [High, for the null ITT result]. In the BRCA-mutated subgroup, rPFS was not reached versus 8.4 months (HR 0.23, 95% CI 0.12–0.43) and OS was not reached versus 23.0 months (HR 0.29, 95% CI 0.14–0.56) [Low]. FDA approval is restricted to BRCA-mutated mCRPC.

TALAPRO-2 (talazoparib plus enzalutamide) reported final OS of 45.8 versus 37.0 months in the all-comer population (HR 0.80, 95% CI 0.66–0.96; *p* = 0.016) [33] [High]. In the HRR-deficient cohort, OS was 45.1 versus 31.1 months (HR 0.62, 95% CI 0.48–0.81), and in the BRCA1/2 subgroup OS was not reached versus 28.5 months (HR 0.50, 95% CI 0.32–0.78; *p* = 0.0017) [Low]. Updated rPFS in the HRR-deficient cohort was 30.7 versus 12.3 months (HR 0.47). The all-comer OS result is the single most important finding in this section, because it is alpha-controlled and demonstrates that benefit was not confined to BRCA-mutated disease.

MAGNITUDE (niraparib plus abiraterone) met futility criteria in the non-BRCA HRR and unselected cohorts, with an rPFS HR of 0.53 in BRCA1/2-mutated disease [34] [Moderate for rPFS; no OS benefit has been established]. FDA approval is restricted to BRCA-mutated mCRPC. MAGNITUDE should not be cited as mature OS evidence.

BRCAAway, a randomised phase II trial comparing abiraterone/prednisone, olaparib and their combination in BRCA1/2- or ATM-altered mCRPC, showed longer PFS with upfront combination therapy than with either single agent [35] [Moderate]. Of 165 eligible patients, 61 with BRCA1/2 or ATM alterations were randomised to abiraterone/prednisone (*n* = 19), olaparib (*n* = 21) or their combination (*n* = 21). Median PFS was 8.6, 14 and 39 months, respectively; the PFS HRs for the combination were 0.33 (95% CI 0.15–0.72) versus abiraterone/prednisone and 0.37 (95% CI 0.17–0.84) versus olaparib [36].

In the ASCO GU 2026 OS update, median OS was 28 months with abiraterone/prednisone, 37 months with olaparib and 68 months with the combination; the combination OS HRs were 0.39 (95% CI 0.16–0.93) versus abiraterone/prednisone and 0.51 (95% CI 0.22–1.18) versus olaparib [36] [Preliminary]. Both comparisons should be considered together: the comparison against olaparib is the more stringent benchmark and did not show a statistically significant OS difference. The trial was small, and its OS analysis was not powered for definitive inference.

The first-line combination trials differed in their inclusion of patients with prior ARPI exposure in the hormone-sensitive setting, limiting generalisation to this population [32,33,34]. Whether the ARPI component still contributes when it represents re-exposure to a failed class is genuinely unknown. The hypothesis that ARPIs induce a “BRCAness” state by suppressing HRR gene expression is a [Mechanistic inference]: it is untested in ARPI-pre-exposed patients and is unlikely to hold once AR-V7 or AR amplification has restored ligand-independent AR signalling. Because prospective evidence for PARPi plus ARPI after prior ARPI exposure in the hormone-sensitive setting is lacking, a clinical trial is the preferred setting for this combination in this population. Where no trial is available, the decision is one for individualised discussion, recognising that the licensed indications do not distinguish by prior ARPI exposure and that the supporting argument against the combination is mechanistic rather than empirical.

### 3.3. PARPi Resistance and Its Sequencing Implications

BRCA reversion mutations are a documented mechanism of acquired PARPi resistance [24,37]. Because platinum agents exploit the same homologous recombination defect, selection for reversions under platinum exposure, and hence cross-resistance, is biologically plausible; its frequency and clinical impact in prostate cancer have not been established [Mechanistic inference]. Other mechanisms described across tumour types include PARP1 mutations that abrogate PARP trapping [38] and restoration of replication-fork protection [39]; the prostate-specific clinical relevance of each is not established [Mechanistic inference].

At progression on a PARPi, ctDNA testing for reversions may be considered where validated assays exist [Low], but its use to guide treatment switching remains investigational; a detected reversion argues against PARPi rechallenge. Because a reversion restores homologous recombination proficiency, it also removes the defect that sensitises the tumour to platinum; detection of a reversion therefore does not provide a biological rationale for preferring platinum-based chemotherapy [Mechanistic inference]. Where PSMA-PET remains positive, ^177^Lu-PSMA-617 is the more defensible next option; where it is not, taxane chemotherapy or trial enrolment applies.

## 4. Taxane Chemotherapy and the CARD Trial

Before turning to radioligand therapy, one trial provides the most direct prospective evidence for treatment sequencing in mCRPC and involves taxane chemotherapy. CARD is highly informative for patients who have already received docetaxel and an ARPI, but its post-docetaxel population limits extrapolation to taxane-naive patients.

### 4.1. CARD: The Strongest Sequencing-Specific Dataset

CARD randomised 255 patients who had progressed on both docetaxel and a prior ARPI to cabazitaxel or to a different ARPI [40]. Cabazitaxel was superior for rPFS (8.0 versus 3.7 months; HR 0.54, 95% CI 0.40–0.73) and for OS (13.6 versus 11.0 months; HR 0.64, 95% CI 0.46–0.89) [High]. The cabazitaxel dose and schedule derive from TROPIC, which established its activity after docetaxel [41].

CARD is the closest that mCRPC has to a true sequencing trial. Two limitations should be stated explicitly and are carried consistently through this review. First, its population was post-docetaxel, so it does not directly answer treatment choice before first exposure to taxane chemotherapy. Second, CARD enrolled patients before ARPI intensification in mHSPC became standard, so few, if any, had received an ARPI in the hormone-sensitive setting (Section 11); the same ARPI-pre-exposure limitation that this review applies to PSMAfore, PROfound and the combination trials applies with equal force to CARD itself. CARD therefore establishes that cabazitaxel outperforms an ARPI switch in patients whose ARPI exposure occurred in the castration-resistant setting, and its extension to patients whose ARPI exposure occurred in mHSPC is an extrapolation, albeit a reasonable one.

### 4.2. Docetaxel Sequencing

Docetaxel (75 mg/m^2^ every three weeks) was the first agent to demonstrate an OS benefit in mCRPC and remains appropriate in chemotherapy-naive patients [2]. Many patients now reach mCRPC having already received docetaxel for mHSPC; a treatment-free interval of at least 12 months may permit rechallenge, but prospective data are limited [Low].

## 5. ^177^Lu-PSMA-617: A Strong rPFS Signal, Confounded Overall Survival, and Comparator Limitations

The evidence for ^177^Lu-PSMA-617 must be interpreted according to the comparator and treatment setting. CARD directly informs post-docetaxel sequencing, whereas PSMAfore enrolled taxane-naive patients and therefore addresses a different clinical population. The rPFS signals are strong. The OS picture is complicated, and the quality of the control arms is relevant to interpreting it.

### 5.1. Post-Taxane: VISION

VISION demonstrated an rPFS of 8.7 versus 3.4 months (HR 0.40) and an OS of 15.3 versus 11.3 months (HR 0.62, 95% CI 0.52–0.74) when ^177^Lu-PSMA-617 was added to standard care in post-ARPI, post-taxane, PSMA-PET-positive mCRPC; the agent was approved by the FDA on 23 March 2022 [42] [High].

An exploratory secondary analysis of 826 VISION participants examined quantitative baseline ^68^Ga-PSMA-11 PET parameters [43]. Whole-body tumour SUVmean was the best single predictor of ^177^Lu-PSMA-617 efficacy, and a one-unit increase was associated with an approximately 12% lower risk of an rPFS event and 10% lower risk of death. Importantly, ^177^Lu-PSMA-617 prolonged both rPFS and OS in every SUVmean quartile, with no identifiable optimum threshold [Low]. This analysis therefore supports SUVmean as a predictor of the *magnitude* of benefit but does not identify a cut-off below which treatment should be withheld, and it should not be cited as evidence that binary PSMA positivity misclassifies patients who do not benefit.

### 5.2. Pre-Taxane Versus ARPI Switch: PSMAfore, SPLASH and ECLIPSE, and Why the Comparator Matters

PSMAfore randomised 468 taxane-naive, post-ARPI, PSMA-positive patients (*n* = 234 per arm) to ^177^Lu-PSMA-617 or an ARPI switch, with randomisation stratified by the setting of the previous ARPI (hormone-sensitive versus castration-resistant) [44]. rPFS favoured RLT (primary analysis 9.3 versus 5.6 months; HR 0.41; updated HR 0.49, 95% CI 0.39–0.61). Final OS showed no significant ITT difference (24.48 versus 23.13 months; HR 0.91, 95% CI 0.72–1.14; *p* = 0.20); 60.3% of patients randomised to the ARPI switch crossed over (75.4% of those with centrally confirmed progression), and the crossover-adjusted OS HR by inverse probability of censoring weighting (IPCW) was 0.59 (95% CI 0.38–0.91). Exposure-adjusted incidences of grade ≥3 treatment-emergent adverse events were 60.8 versus 85.1 per 100 patient-treatment years, and of serious events 32.5 versus 49.9 [Moderate for rPFS; High for the null ITT OS].

The IPCW estimate should be interpreted with caution: it assumes no unmeasured confounding of the crossover decision, an assumption the investigators acknowledge cannot be verified. A rank-preserving structural failure time (RPSFT) adjustment was also performed, but the investigators judged its common-treatment-effect assumption unlikely to hold in the presence of overlapping ITT survival curves, and they concluded that the IPCW analysis identified a benefit that would not have been detected by either the ITT or the RPSFT analysis [44]. The adjusted estimate is therefore model-dependent and hypothesis-generating.

SPLASH (^177^Lu-PNT2002) also reported an rPFS benefit over an ARPI switch in post-ARPI, taxane-naive, PSMA-positive disease [45] [Preliminary], and ECLIPSE (^177^Lu-PSMA-I&T) was reported by its sponsor to have met its rPFS primary endpoint against the same comparator [46] [Preliminary]. Neither has yet been published in full.

Each of these three trials used an ARPI switch as its control. The clinical value of that comparator is limited relative to chemotherapy in patients who have already progressed on an ARPI, and CARD, PLATO and the Khalaf crossover trial each bear on this, with the qualifications set out in Section 1 and Section 4.1: CARD tested the comparison only after docetaxel, PLATO tested combination continuation rather than a switch, and Khalaf found asymmetric activity, with a 36% PSA response rate for enzalutamide after abiraterone. Taken together these do not establish that an ARPI switch is without value, but they do establish that it is a weak comparator against which to demonstrate superiority. Demonstrating an rPFS benefit over such a comparator establishes that RLT outperforms a comparator of limited clinical value, not that it outperforms the best available option. This is why the head-to-head comparisons against taxanes carry more practical weight.

### 5.3. Head-to-Head Against Taxanes: TheraP and PLUDO/PR.21

TheraP, a randomised phase II trial in post-docetaxel mCRPC (*n* = 200), compared ^177^Lu-PSMA-617 with cabazitaxel. PSA50 responses were more frequent with RLT (66% versus 37%), and grade 3–4 adverse events less frequent (33% versus 53%) [47]; rPFS also favoured RLT. With a median follow-up of 35.7 months, however, OS was similar between arms (restricted mean survival time 19.1 versus 19.6 months; *p* = 0.77) [48] [Moderate]. Deeper PSA responses therefore did not translate into longer survival.

PLUDO (CCTG PR.21), a randomised phase II trial, compared ^177^Lu-PSMA-617 with docetaxel in 199 chemotherapy-naive patients with PSMA-PET-positive mCRPC progressing after ARPI therapy [13]. The primary endpoint was not met: median rPFS was 8.6 versus 10.7 months (HR 1.02, 90% CI 0.77–1.35) [13]. ^177^Lu-PSMA-617 produced more PSA50 responses (53% versus 32%) and fewer grade 3–4 adverse events (13% versus 34%) [13]. Median intention-to-treat OS was 14.3 months with ^177^Lu-PSMA-617 and 18.2 months with docetaxel (HR 1.64, 90% CI 1.14–2.35; two-sided *p* = 0.02) [13]. Crossover was asymmetric: 62 patients moved from docetaxel to ^177^Lu-PSMA-617, and 40 in the reverse direction [13] [Preliminary].

This OS result should not be understated. It is the only randomised OS comparison of RLT with a taxane in chemotherapy-naive, post-ARPI disease, and its point estimate favours docetaxel first. In a crossover analysis presented at ASCO 2026, rPFS and OS were similar irrespective of sequence among patients who received both agents [49] [Preliminary]. Because that analysis is conditional on a post-randomisation event, surviving and remaining fit enough for a second agent, it loses the protection of randomisation and is exposed to survivorship bias and confounding by indication. It is consistent with the intention-to-treat difference reflecting asymmetric crossover, but it cannot demonstrate this, and it cannot establish that the two sequences are equivalent.

Taken together, TheraP and PLUDO provide evidence on RLT versus taxanes in *different* treatment populations: TheraP enrolled post-docetaxel patients, whereas PLUDO enrolled chemotherapy-naive patients. They are therefore complementary rather than confirmatory and should not be described as comparable populations. Both showed higher PSA response with RLT; neither established an OS advantage for RLT, and PLUDO’s point estimate favoured docetaxel first. This supports individualised shared decision-making between RLT and taxane chemotherapy on the basis of patient priorities and tolerability, while recognising that the only randomised OS signal in the chemotherapy-naive setting currently favours docetaxel first and requires confirmation.

### 5.4. The Hormone-Sensitive Setting: PSMAddition

PSMAddition randomised 1144 patients with PSMA-positive mHSPC to ^177^Lu-PSMA-617 plus ADT and an ARPI or to ADT and an ARPI alone, and met its primary rPFS endpoint (HR 0.72, 95% CI 0.58–0.90; *p* = 0.0021) [12] [Moderate]. Grade ≥ 3 adverse events occurred in 51% versus 43% of patients [12]. Interim OS was immature and not statistically significant (HR 0.84) [50] [Preliminary]; 59.9% of control patients with confirmed radiographic progression crossed over, approximately 16% of the control arm. If an OS benefit is confirmed, RLT will migrate into the hormone-sensitive setting and further compress the options available at the mCRPC transition.

### 5.5. PSMA Biology and Heterogeneity

PSMA-PET eligibility is not uniform across trials. VISION excluded approximately 13% of screened patients on imaging grounds [42], whereas the stricter TheraP criteria, which also excluded FDG-discordant disease, excluded 27% (80 of 291) of registered men after PET [48] [Low]. PSMA expression is upregulated by ADT and suppressed both by prolonged ARPI exposure, through promoter hypermethylation, and by neuroendocrine transdifferentiation [51]. A speculative link between BRCA2 loss and neuroendocrine differentiation raises the hypothesis that the patients most likely to benefit from a PARPi may also be at higher risk of losing PSMA expression and therefore RLT eligibility, although this remains entirely a [Mechanistic inference].

Because eligibility thresholds differ across trials, “PSMA-positive” is not a single entity. In this review the term denotes eligibility by the criteria of the trial being discussed, and the framework in Section 9 should be read accordingly.

### 5.6. Radioligand Therapy Combinations

LuPARP (^177^Lu-PSMA-617 plus olaparib) produced a PSA50 rate of approximately 62% in a phase I study [52] [Preliminary], with myelosuppression as the principal dose-limiting concern. In the randomised phase II ENZA-p trial, the primary readout showed a significant improvement in PSA-PFS (median 13.0 versus 7.8 months; HR 0.43, 95% CI 0.29–0.63) [53] [Moderate]. A subsequent OS analysis favoured the combination (median 34 versus 26 months; HR 0.55, 95% CI 0.36–0.84; *p* = 0.0053) [54] [Preliminary], despite 38% crossover to ^177^Lu-PSMA-617 in the control arm. ENZA-p enrolled an ARPI-naive, poor-risk population selected for features predicting early enzalutamide failure. This population differs materially from the ARPI-pre-exposed population that is the focus of this review, and the results cannot be extrapolated to the post-ARPI setting. These combination data therefore remain insufficient to establish an optimal treatment sequence in ARPI-pre-exposed mCRPC.

## 6. Antibody–Drug Conjugates: Where the Platform Stands

For patients who have exhausted or are ineligible for RLT, particularly those with PSMA-low or PSMA-negative disease after prior therapy, antibody–drug conjugates represent the next investigational frontier. The platform carries early promise; it does not yet carry phase III evidence.

### 6.1. Design and Prostate-Specific Challenges

ADCs link a monoclonal antibody to a cytotoxic payload, predominantly a topoisomerase I inhibitor such as DXd or SN-38, through a cleavable linker. Prostate cancer poses specific problems: an immunologically cold microenvironment, heterogeneous antigen expression within and between tumours, and selection for antigen-low escape clones under prior ARPI and RLT exposure [55,56].

### 6.2. Active Targets and Clinical Evidence

PSMA-directed ADCs have achieved PSA50 rates of approximately 14% in heavily pre-treated mCRPC, substantially below ^177^Lu-PSMA-617, plausibly because antibody internalisation kinetics are inferior to those of the small-molecule ligand [55] [Low].

STEAP1 may provide an alternative target in some PSMA-low tumours, although its expression can decline with neuroendocrine differentiation [56]. Its potential use after RLT remains a [Mechanistic inference].

B7-H3 (CD276) is broadly expressed across adenocarcinoma and neuroendocrine subtypes and is negatively regulated by AR signalling [57]. In a phase I/II study of DB-1311/BNT324 (NCT05914116), 73 heavily pre-treated patients with mCRPC were enrolled [58]. Among the 52 response-evaluable patients with measurable disease at baseline, the unconfirmed objective response rate was 42.3% (22/52; 95% CI 28.7–56.8) and the disease-control rate 90.4% (47/52; 95% CI 79.0–96.8); among 68 evaluable patients, the six-month rPFS rate was 67.7% [58] [Preliminary].

DLL3 is selectively overexpressed in treatment-emergent neuroendocrine prostate cancer (NEPC); no DLL3-directed agent is yet approved for prostate cancer, and phase I trials are ongoing [59] [Mechanistic inference].

ABBV-969, a bispecific PSMA × STEAP1 ADC, was reported at ASCO 2026 in 49 heavily pre-treated patients (median five prior lines; 84% prior docetaxel; 47% prior ^177^Lu-PSMA-617). At active dose levels, PSA50 and PSA90 response rates were 67% and 28%, respectively, and the confirmed ORR was 45% among 29 RECIST-evaluable patients [14] [Preliminary]. These preliminary results support further investigation but do not establish efficacy specifically in the prior-RLT subgroup or demonstrate activity against PSMA-loss escape clones.

### 6.3. Antigen Coverage and the Bispecific Rationale

Immunofluorescence of mCRPC tissue microarrays indicates that B7-H3 and STEAP1 together are expressed on approximately 82% of mCRPC cells, more than any single antigen, leaving roughly 18% double-negative [57,60] [Low]. This is the biological rationale for bispecific ADC design. These tissue-expression estimates are not clinical response predictors and have not been validated as such.

## 7. Treatment-Emergent Neuroendocrine Prostate Cancer

NEPC is the terminal resistance phenotype for most approved mCRPC agents and has no approved disease-specific therapy.

### 7.1. Prevalence and Timing

Pure NEPC occurs in fewer than 1% of prostate cancers at diagnosis, but treatment-emergent small-cell neuroendocrine prostate cancer was identified in 17% of patients with mCRPC in a prospective multi-institutional biopsy study [61] [Low]. Series examining late-stage castration-resistant disease, including rapid-autopsy cohorts, have identified neuroendocrine features in a substantial minority of cases, with reported frequencies varying widely according to the definition used, treatment history and tissue source [62] [Low]. Most cases emerge after two or more years of ARPI exposure.

### 7.2. Molecular Architecture

Co-deletion of TP53 and RB1 disrupts cell-cycle checkpoints, permits lineage infidelity, and is enriched in NEPC relative to castration-resistant adenocarcinoma [62] [Low]. AURKA and MYCN amplification drive neuroendocrine transcription through N-Myc target genes. CDK5 is activated by binding its regulatory subunit p35, which is transcriptionally upregulated downstream of MYCN; activated CDK5 phosphorylates RB1 and releases E2F1, reinforcing neuroendocrine differentiation [63]. EZH2 silences luminal epithelial genes through H3K27 methylation and is a core epigenetic driver of the adenocarcinoma-to-NEPC transition; EZH2 inhibition restores androgen receptor expression and antiandrogen sensitivity in lineage-plastic models [64] [Mechanistic inference].

### 7.3. PSMA Loss and ADC Target Implications

AR drives PSMA transcription, so the shift to an AR-low or AR-negative neuroendocrine phenotype suppresses PSMA expression [51]; this is the biological basis for PSMA-PET negativity in NEPC. STEAP1 also declines, although less sharply, whereas B7-H3 and DLL3 remain expressed and DLL3 may be upregulated, which is why they are the principal ADC targets in neuroendocrine-differentiated disease [57,59].

### 7.4. Clinical Detection and Management

NEPC should be suspected in the presence of rapid PSA-discordant progression, elevated serum neuron-specific enolase (NSE) or chromogranin A, or visceral tropism disproportionate to bone burden. The use of ctDNA testing for AURKA and MYCN amplification and TP53/RB1 alterations to detect neuroendocrine transformation remains investigational; synaptophysin and chromogranin A immunohistochemistry on a progressive lesion, preferably visceral, is useful for classification.

The evidence for platinum-based chemotherapy in this setting is limited. In the GETUG P01 phase II study of carboplatin plus etoposide in anaplastic progressive mCRPC, the objective response rate was 8.9% among the 46 patients with measurable disease and the PSA response rate was 8%, with neuroendocrine marker responses in 31% (NSE) and 7% (chromogranin A); grade 3–4 neutropenia occurred in 65.5% of the 55 treated patients, and there was one toxicity-related death. The investigators concluded that the benefit–risk ratio of the regimen was unfavourable [65]. A separate phase II programme of carboplatin–docetaxel followed by cisplatin–etoposide in variant castration-resistant disease reported progression-free rates of 65.4% (74/113) and 33.8% (24/71) after four cycles of first- and second-line therapy, respectively [66]. These are not the only platinum data in this setting, and neither study should be described as the only prospective trial of platinum in NEPC. Platinum-based chemotherapy remains a reasonable option in the absence of an open trial, but it should be offered with explicit acknowledgement of modest activity and substantial haematological toxicity, not on the basis of response rates extrapolated from small-cell lung cancer [Low]. No phase III evidence supports any agent specifically in treatment-emergent NEPC.

### 7.5. Sequencing Implications

By the time NEPC is established, AR-directed and PSMA-directed options may be substantially limited. The opportunity lies earlier, in detecting neuroendocrine differentiation while AR activity and PSMA expression remain partially intact. Mevrometostat plus enzalutamide has generated a randomised phase 1b/2 signal [67] [Preliminary], but the relevant phase III programmes differ by prior ARPI exposure: MEVPRO-1 (NCT06551324) evaluates patients with prior abiraterone exposure, whereas MEVPRO-2 (NCT06629779) enrols ARPI-naive patients. The phase 1b/2 expansion that included abiraterone-pre-exposed patients is therefore the logical antecedent of MEVPRO-1, not MEVPRO-2. Neither trial establishes a sequencing strategy yet.

## 8. Circulating Tumour DNA and Liquid Biopsy: Toward Adaptive Sequencing

### 8.1. Current Clinical Use

ctDNA-based HRR testing at mCRPC diagnosis is the most validated liquid-biopsy application in this disease and provides a complementary alternative to tissue biopsy that is sometimes preferable in bone-dominant disease [68] [Moderate]. Concordance with tissue is high for BRCA2 and acceptable for ATM.

### 8.2. BRCA Reversion Mutations

BRCA reversions restore homologous recombination function and are detectable in ctDNA at progression on a PARPi [24,37] [Low]. A detected reversion indicates that homologous recombination has been restored in a resistant clone, making further PARPi unlikely to help. Because the same restoration removes the basis for platinum sensitivity, a reversion does not provide a rationale for preferring platinum-based chemotherapy, and it argues against PARPi rechallenge. Reversion testing may be considered where validated assays exist, but its use to guide treatment switching remains investigational: no prospective trial has yet shown that ctDNA-guided switching improves outcomes over clinically guided switching.

### 8.3. AR-V7 as a Sequencing Biomarker

Detection of AR-V7 in circulating tumour cells is associated with poor response to abiraterone or enzalutamide [17] [Low]. This is a prospective biomarker association study without validation as a treatment-selection tool, and it is graded accordingly wherever it appears in this review. Routine AR-V7 testing to direct sequencing remains investigational, CTC assay availability is limited, and its role in selecting patients for PARPi plus ARPI combinations is not established.

### 8.4. TP53/RB1 Co-Alteration and NEPC Risk

ctDNA profiling, including TP53 and RB1 alterations, can recognise transformation to neuroendocrine disease [69] [Low]. Serial monitoring for a rising co-alteration fraction alongside NSE may detect neuroendocrine differentiation before clinical signs appear, extending the window for enrolment in NEPC-directed trials. This monitoring strategy remains investigational.

### 8.5. The Adaptive Sequencing Vision

The direction of travel is sequential ctDNA assessment at defined intervals feeding real-time adjustments to sequencing: a reversion triggers a switch away from a PARPi; rising AR amplification flags impending ARPI failure; rising NSE with AURKA amplification signals early neuroendocrine transition. This model is investigational and should not be implemented outside a research protocol.

## 9. A Hypothesis-Generating Conceptual Sequencing Framework

The framework below organises the available evidence into four patient tracks stratified by HRR status and PSMA-PET expression (Figure 2). It is explicitly conceptual and provisional: it is not a guideline, not a consensus statement and not validated by any prospective sequencing trial. Clinicians and patients should treat these tracks as hypothesis-generating starting points for individualised shared decision-making rather than as prescriptive algorithms. Performance status, symptom burden, marrow reserve, prior toxicity, clinical trial availability and patient preference can and should override any algorithmic recommendation. The track prevalences are approximate, derived from separate cohorts, and are not intended to sum to 100%.

Guideline grounding. The framework treats ARPI-to-ARPI sequencing as generally disfavoured after progression on an ARPI. The EAU guideline advises against sequencing of androgen-receptor-targeted agents as a weak recommendation [11], and supporting evidence comes from three sources, with the limitations noted in Section 1, Section 4.1 and Section 5.2: CARD showed superior overall survival for cabazitaxel over an ARPI switch after docetaxel and prior ARPI (HR 0.64) [40], PLATO found no benefit from continuing enzalutamide alongside abiraterone [10], and the Khalaf crossover trial showed PSA responses in only 4% of patients given abiraterone after enzalutamide, though 36% responded to enzalutamide after abiraterone [9]. Post-ARPI treatment should therefore be directed by molecular profiling, PARP inhibitors for HRR-altered disease and ^177^Lu-PSMA-617 for PSMA-positive disease, or by taxane chemotherapy. The tracks that follow operate within that constraint.

Assessments informing the framework, with their current status. Guideline-supported: comprehensive HRR profiling, germline and somatic, with ctDNA supplementation where tissue is inadequate; PSMA-PET where radioligand therapy is contemplated, recognising that eligibility thresholds differ across trials; and MSI-H/dMMR testing, since pembrolizumab has shown clinical efficacy in selected patients with MSI-H/dMMR mCRPC and is supported by tissue-agnostic evidence [28,70]. Investigational, and not required for routine mCRPC management: AR-V7 in circulating tumour cells; ctDNA co-alteration status (TP53/RB1); and baseline or serial BRCA reversion testing. These investigational assessments should be undertaken within a research protocol wherever possible (Section 8.5).

### 9.1. Track A: BRCA1/2-Mutated, PSMA-Positive (Approximately 8–10%)

This track applies to BRCA2 and BRCA1 alterations. Patients with BRCA1 mutations may follow the same path with a lower expected response magnitude, on the basis of the subgroup data in Section 2.2 [Low].

One reasonable evidence-informed approach is PARPi-based therapy first. In ARPI-naive patients, a PARPi plus ARPI combination (olaparib plus abiraterone, talazoparib plus enzalutamide, or niraparib plus abiraterone) carries the most mature evidence. The strength of that evidence should be stated precisely: TALAPRO-2 demonstrated an alpha-controlled all-comer OS benefit [High]; the larger BRCA-specific estimates from PROpel and TALAPRO-2 are exploratory [Low]; and MAGNITUDE has demonstrated an rPFS benefit in BRCA-mutated disease but no OS benefit [Moderate]. Niraparib plus abiraterone should therefore not be described as supported by mature OS evidence.

In ARPI-pre-exposed patients, now the majority, olaparib or rucaparib monotherapy has the most directly applicable evidence [High for PROfound cohort A OS; Moderate for TRITON3 rPFS]. Combination therapy in this setting is extrapolated from ARPI-naive trials; a clinical trial is the preferred setting, and where none is available the decision should be individualised rather than categorically excluded, since the argument against it is mechanistic [Mechanistic inference].

At progression: investigational ctDNA reversion testing may be considered where validated assays are available [Low]. If no reversion is detected, ^177^Lu-PSMA-617 can be used where PSMA-PET remains positive. If a reversion is detected, PARPi rechallenge is unlikely to help, and reversion status alone does not justify preferentially selecting platinum, because restored homologous recombination can confer platinum resistance; ^177^Lu-PSMA-617 where PSMA-PET remains positive, or cabazitaxel, are the more defensible options. Platinum is not contraindicated on the basis of reversion status; it is simply not preferentially indicated. For BRCA2 homozygous deletion, enrolment in a LuPARP-type combination trial merits consideration, since deletion precludes reversion [Mechanistic inference].

Subsequent lines: cabazitaxel, informed by CARD [High], subject to the post-docetaxel qualification in Section 4.1; ABBV-969 or a B7-H3 ADC trial in the event of PSMA-low escape [Preliminary].

### 9.2. Track B: Non-BRCA1/2 HRR-Mutated, PSMA-Positive (Approximately 4–8%)

The prevalence stated here is derived as follows: HRR alterations occur in approximately 20–25% of mCRPC, of which BRCA1/2 accounts for approximately 15%, leaving approximately 5–10% non-BRCA HRR; applying the proportion of patients who are PSMA-PET-positive gives approximately 4–8%.

ATM-mutated disease: PARPi activity in ATM-altered disease is substantially less consistent than in BRCA-altered disease, and in TRITON3 the ATM subgroup derived no rPFS benefit [Low]. Where PSMA uptake is high, ^177^Lu-PSMA-617 first is a reasonable alternative. There are no head-to-head data; performance status and patient preference should guide the choice.

CDK12 biallelic loss: evidence for PARPi monotherapy benefit remains limited and uncertain [Low]. PROfound cohort B, which included CDK12-altered patients, did not establish a gene-specific benefit, and current ASCO recommendations for PARPi are anchored in BRCA1/2 alterations [15,28]; treatment should therefore be individualised rather than categorically excluded. This is a case for individualised consideration, not a categorical recommendation to avoid PARPi. ^177^Lu-PSMA-617 is appropriate if PSMA-positive, although the supporting evidence rests on PSMA positivity rather than on CDK12 status, since no CDK12-defined subgroup was prospectively evaluated in VISION [Moderate]; otherwise, a bispecific T-cell engager or checkpoint-inhibitor trial is appropriate, given the genomic features associated with CDK12 loss [29] [Mechanistic inference]. Trial enrolment is the priority.

CHEK2, PALB2 and BRIP1: trial enrolment where possible. For PALB2, a PARPi may be considered [Low]; for monoallelic CHEK2, substantially less benefit should be expected [Mechanistic inference].

### 9.3. Track C: HRR Wild-Type, PSMA-Positive (Approximately 50–60%)

This is the largest group and the one in which the comparator critique matters most. RLT has a strong rPFS signal against an ARPI switch (PSMAfore) but no proven OS advantage over a taxane, and in the only randomised chemotherapy-naive comparison the OS point estimate favoured docetaxel first (PLUDO) [Preliminary].

A defensible approach is ^177^Lu-PSMA-617 or docetaxel as the first post-ARPI therapy, with the choice driven by disease phenotype and patient priorities. The two options do not carry equal guideline support, and this should be stated openly: current ASCO guidance recommends docetaxel for patients who have received a prior ARPI, reserving ^177^Lu-PSMA-617 or cabazitaxel for those who have received both a prior ARPI and docetaxel [28]. Offering RLT in the taxane-naive setting is supported by the PSMAfore rPFS result and by regulatory approval in some jurisdictions, but it represents a divergence from that guideline recommendation, and the divergence should be explicit in the consent discussion. The choice should account for disease tempo, symptoms, prior therapy, eligibility, toxicity and patient preference [Moderate].

TheraP and PLUDO do not establish an optimal order in either direction; neither was designed to answer the sequencing question, and the PLUDO crossover analysis is conditioned on a post-randomisation subset. Sequencing may therefore reasonably follow tolerability and patient preference, but this reflects the absence of definitive evidence rather than demonstrated equivalence [Moderate], and the direction of the PLUDO ITT OS result should be disclosed rather than set aside.

After the first agent: the alternative of RLT or taxane; cabazitaxel if docetaxel has already been used, informed by CARD [High]. Later lines: ABBV-969 or a B7-H3 ADC trial in the event of PSMA-low escape [Preliminary]; a ^225^Ac-PSMA-617 trial where PSMA is retained after ^177^Lu [Preliminary]; radium-223 for symptomatic bone-only disease [71] [High].

PEACE-3 demonstrated an OS benefit for enzalutamide plus radium-223 over enzalutamide alone in first-line mCRPC (median OS 38.2 versus 32.6 months; HR 0.76, 95% CI 0.60–0.96; *p* = 0.0096; rPFS HR 0.71, 95% CI 0.57–0.89) in a largely ARPI-naive population [72] [High]. Its extrapolation to patients previously treated with an ARPI is graded [Moderate]. Grade ≥3 treatment-emergent adverse events were more frequent with the combination (69.3% versus 57.6%). Co-administration of a bone-protecting agent became mandatory for all patients in March 2018 and is required with this combination [72]. Radium-223 should not be combined with abiraterone plus prednisone, on the basis of ERA-223, which reported excess fractures and an unfavourable mortality signal [73].

### 9.4. Track D: PSMA-Negative or Heterogeneous (Approximately 15–25% of Screened Patients)

The first step is evaluation for NEPC: serum NSE and chromogranin A, tissue assessment where clinically indicated, and investigational ctDNA assessment for AURKA/MYCN amplification and TP53/RB1 co-alteration. Re-biopsy of a progressive PSMA-negative lesion should be considered when the result would alter treatment. Where available, FDG-PET may be used alongside PSMA-PET to identify discordant, PSMA-negative but FDG-avid disease, a pattern associated with poor outcome after RLT; this use is not standardised across trials and is not a requirement for routine management [Low].

Where NEPC is confirmed, one reasonable evidence-informed option is a DLL3-targeting ADC trial where available [Preliminary]; platinum plus etoposide if no trial is open, with the activity and toxicity caveats set out in Section 7.4 [Low]; or a B7-H3 ADC trial [Preliminary].

In AR-positive, PSMA-negative disease: a PARPi if BRCA1/2-mutated [High for PROfound cohort A; Low for the BRCA subgroup estimate], noting that for non-BRCA HRR alterations the evidence is that of Track B [Low] rather than [High]; docetaxel [High]; a B7-H3 ADC trial [Preliminary]; or an AR-degrader trial if an actionable AR ligand-binding-domain alteration is detected on ctDNA [Preliminary]. Repeat PSMA-PET after starting an AR-directed therapy should be undertaken only within an investigational strategy; recovery of PSMA expression is a mechanistic hypothesis without prospective validation [Mechanistic inference].

## 10. Evidence Summary with Major Caveats

Table 2 summarises the preferred option for each clinical scenario discussed above, with the supporting evidence level, the key data and the principal caveat attached to each recommendation. Evidence levels in this table are those defined in Table 1 and are identical to those used in the corresponding text sections and in Figure 2 and Figure 3.

## 11. Key Trial Comparison

Table 3 summarises the pivotal trials referenced throughout this review, with explicit notation of each comparator arm, the ARPI-exposure setting of the enrolled population and the principal limitation of each trial. The ARPI-exposure column is clinically important, because it shows that most of the evidence base was generated in populations that do not represent contemporary patients who received an ARPI for mHSPC.

ARPI-exposure setting is taken from each trial’s eligibility criteria. The proportion of patients whose prior ARPI was given for mHSPC was small or not separately reported in most trials; only PSMAfore stratified randomisation by prior-ARPI setting. * Randomised phase II. ADT, androgen-deprivation therapy; ARPI, androgen receptor pathway inhibitor; CI, confidence interval; HR, hazard ratio; HRR, homologous recombination repair; IPCW, inverse probability of censoring weighting; ITT, intention to treat; mHSPC, metastatic hormone-sensitive prostate cancer; NS, not significant; OS, overall survival; rPFS, radiographic progression-free survival.

## 12. Discussion: Critical Appraisal and Evidence Gaps

Three critical observations follow from the evidence assembled above, and together they define what this review can and cannot support.

First, the comparator problem. Every randomised trial of radioligand therapy against an ARPI switch measured benefit against a control arm of limited clinical value in patients already progressing on an ARPI. A demonstrated rPFS advantage therefore establishes superiority over a comparator of limited clinical value rather than over the best available option. This argument should be stated with its own limitations attached: CARD tested the ARPI switch only after docetaxel, PLATO tested combination continuation rather than a switch, and Khalaf found that enzalutamide after abiraterone retained a 36% PSA response rate. The comparator is weak; it is not worthless, and describing it as obsolete would exceed what these three trials establish.

Second, the population problem. Prior ARPI exposure during mCRPC should be distinguished from exposure in the hormone-sensitive setting; evidence from the former does not directly establish the optimal treatment sequence after the latter. This limitation applies uniformly, including to CARD, which enrolled patients before ARPI use in mHSPC became standard.

Third, the endpoint problem. The agents with the strongest rPFS and PSA-response signals are not those with demonstrated overall survival advantages over active comparators, and depth of response has repeatedly failed to translate into survival gain. TheraP and PLUDO are the clearest illustrations.

These three observations, rather than any individual trial result, are the substantive contribution of this review, and they are the reason the framework in Section 9 is presented as hypothesis-generating rather than directive. The evidence landscape is visualised in Figure 3, which plots the major trials by evidence level and sequencing relevance. The upper-right zone, comprising high-quality evidence from a randomised comparison of two post-ARPI strategies in an unselected population, contains only CARD.

No prospective sequencing trial has been conducted. Every pathway proposed in Section 9 rests on single-arm studies, phase II data, mechanistic inference, or extrapolation to populations not represented in the pivotal trials.

The PARPi plus ARPI combination data, although now mature for OS in the all-comer population of TALAPRO-2, provide limited evidence specifically for patients previously exposed to an ARPI in the hormone-sensitive setting. The BRCA-specific estimates that dominate clinical discussion are exploratory, and the null BRCA OS result in TRITON3 shows that they are not uniformly reproducible.

Randomised trials of RLT against an ARPI switch demonstrate rPFS benefit in PSMA-positive, taxane-naive populations, but the clinical interpretation of the comparator differs from head-to-head taxane trials. PSMAfore had substantial crossover and no significant ITT OS difference; SPLASH and ECLIPSE used the same ARPI-switch comparator and have so far been reported only in congress or topline form [45,46]. The head-to-head taxane trials provide complementary evidence in different treatment populations, and the only randomised OS comparison in the chemotherapy-naive setting favoured docetaxel first.

PSMA quantification is not standardised, and binary positivity does not capture the SUVmean variation that predicts the magnitude of RLT benefit. The VISION exploratory PET analysis nonetheless found benefit across all SUVmean quartiles, so quantification refines expected benefit rather than identifying a group that cannot benefit.

No agent has been approved specifically for treatment-emergent NEPC, and ctDNA-guided adaptive switching has not been evaluated prospectively.

Access and implementation. The framework assumes access to comprehensive HRR profiling, PSMA-PET, ctDNA testing and radioligand therapy, which is not uniform across health systems, including many middle-income settings. Where somatic profiling is unavailable, sequencing must rely on clinical phenotype, supplemented by germline testing where possible. Where PSMA-PET is inaccessible, eligibility for PSMA-targeted RLT cannot be established locally. Serial ctDNA monitoring to trigger treatment changes remains investigational, whereas ctDNA-based genomic profiling can support treatment selection when tissue is inadequate [68]. Where radioligand therapy is unavailable, docetaxel followed by cabazitaxel, anchored in CARD [40], remains a fully evidence-based sequence rather than a second-best compromise. Resource limitations may therefore restrict the available pathways and require adaptation to local diagnostic and treatment access.

Cumulative toxicity constrains any theoretical sequence: many patients will not remain fit for four or five sequential agents. The framework should therefore be read as an evidence map rather than an algorithm, with prior treatment, symptoms, disease tempo, molecular profile, PSMA expression, toxicity, access and patient preference determining treatment choice.

## 13. Future Directions

### 13.1. AR Degraders: rechARge

rechARge (phase III, NCT06764485) compares BMS-986365 with investigator’s choice in post-ARPI mCRPC [74]. Early clinical activity has been reported with other AR-directed degraders, including bavdegalutamide (ARV-110) [76] [Preliminary]. This provides proof of concept for the class but should not be used to infer the efficacy of BMS-986365, which is a structurally distinct agent, before its phase III results are available.

### 13.2. EZH2 Inhibition: MEVPRO-1 and MEVPRO-2

Mevrometostat plus enzalutamide showed an rPFS of 14.3 versus 6.2 months (HR 0.51; 90% CI 0.28–0.95) in a randomised phase 1b/2 expansion that included abiraterone-pre-exposed patients [67] [Preliminary]. The confidence interval is wide and approaches unity. MEVPRO-1 (NCT06551324) is the phase III programme evaluating patients with prior abiraterone exposure and is therefore the trial to which this signal most directly applies; MEVPRO-2 (NCT06629779) enrols ARPI-naive patients. Neither has yet established an optimal sequence.

### 13.3. Alpha Emitters: AcTFirst and PSMAcTION

Phase I data for ^225^Ac-PSMA-617 presented at ASCO 2026 suggested greater activity and better tolerability before rather than after exposure to ^177^Lu-PSMA-617 [77] [Preliminary]; the AcTFirst and PSMAcTION phase III trials will determine whether the alpha emitter should precede or replace beta-emitter RLT.

### 13.4. T-Cell Engagers and Immunotherapy Combinations

In a phase I study of the PSMA × CD3 bispecific T-cell engager pasotuxizumab, PSA declines greater than 50% occurred in 9 of 31 patients receiving subcutaneous treatment and 3 of 16 receiving continuous intravenous treatment [78] [Low]. CDK12-altered mCRPC has genomic features—focal tandem duplications and increased neoantigen burden—that provide a biological rationale for enrolling these patients systematically in bispecific and checkpoint-combination trials [29] [Mechanistic inference]. Although CDK12 loss has been described as defining an immunogenic class of prostate cancer [29], MSI-H/dMMR disease carries a higher mutational and neoantigen burden and remains the only molecular subgroup with demonstrated clinical responses to checkpoint blockade [70].

### 13.5. The Trials the Field Needs

Four studies would resolve most of the uncertainty described in this review: a randomised sequencing trial of PARPi-first versus RLT-first in BRCA2-altered, PSMA-positive disease; a randomised comparison of RLT against docetaxel powered for OS in ARPI-pre-exposed, chemotherapy-naive patients, which PLUDO makes more rather than less urgent; a registrational trial of any agent in treatment-emergent NEPC; and a randomised trial of ctDNA-guided adaptive switching against clinically guided switching.

## 14. Conclusions

Sequencing in mCRPC in 2026 remains a problem with more options than answers, and the evidence supports several cautious conclusions.

ARPI rechallenge after ARPI failure has consistently underperformed, with the asymmetry noted in Section 1: CARD, PLATO, the Khalaf crossover trial and the 2025 EAU guidelines point in the same direction, though none tested the question in the contemporary ARPI-pre-exposed population.

Cabazitaxel after docetaxel and prior ARPI is supported by the OS endpoint of CARD, the clearest sequencing evidence the field possesses.

PARPi-based therapy has its most consistent signal in BRCA1/2-mutated disease, but the two alpha-controlled OS results in the class are PROfound cohort A (HR 0.69) and the TALAPRO-2 all-comer population (HR 0.80). The larger BRCA-specific estimates are exploratory, and the null BRCA OS result in TRITON3 shows they are not uniform. The BRCA OS evidence should therefore be described as promising and internally inconsistent, not as extensive and consistent.

^177^Lu-PSMA-617 has a strong rPFS signal but, measured against the more clinically informative comparator of a taxane, no proven OS advantage; its real strengths are PSA response and tolerability.

Two cautions are warranted. First, the rPFS gains shown against an ARPI switch may not reflect the practical value of RLT, because that comparator is weak in this population. Second, evidence supporting PARPi and RLT after prior ARPI treatment in the castration-resistant setting does not necessarily establish their optimal sequence after ARPI exposure in the hormone-sensitive setting. Sequencing decisions in this population therefore still require extrapolation.

Beyond applying these evidence-informed principles, the most useful action available to clinicians is enrolling eligible patients in sequencing trials, above all in BRCA2-positive, PSMA-positive disease, where the sequencing question is most pressing and least studied. An approved NEPC-specific therapy and a validated ctDNA-guided adaptive sequencing strategy are the two advances that would most change this field.

## Figures and Tables

**Figure 1:**
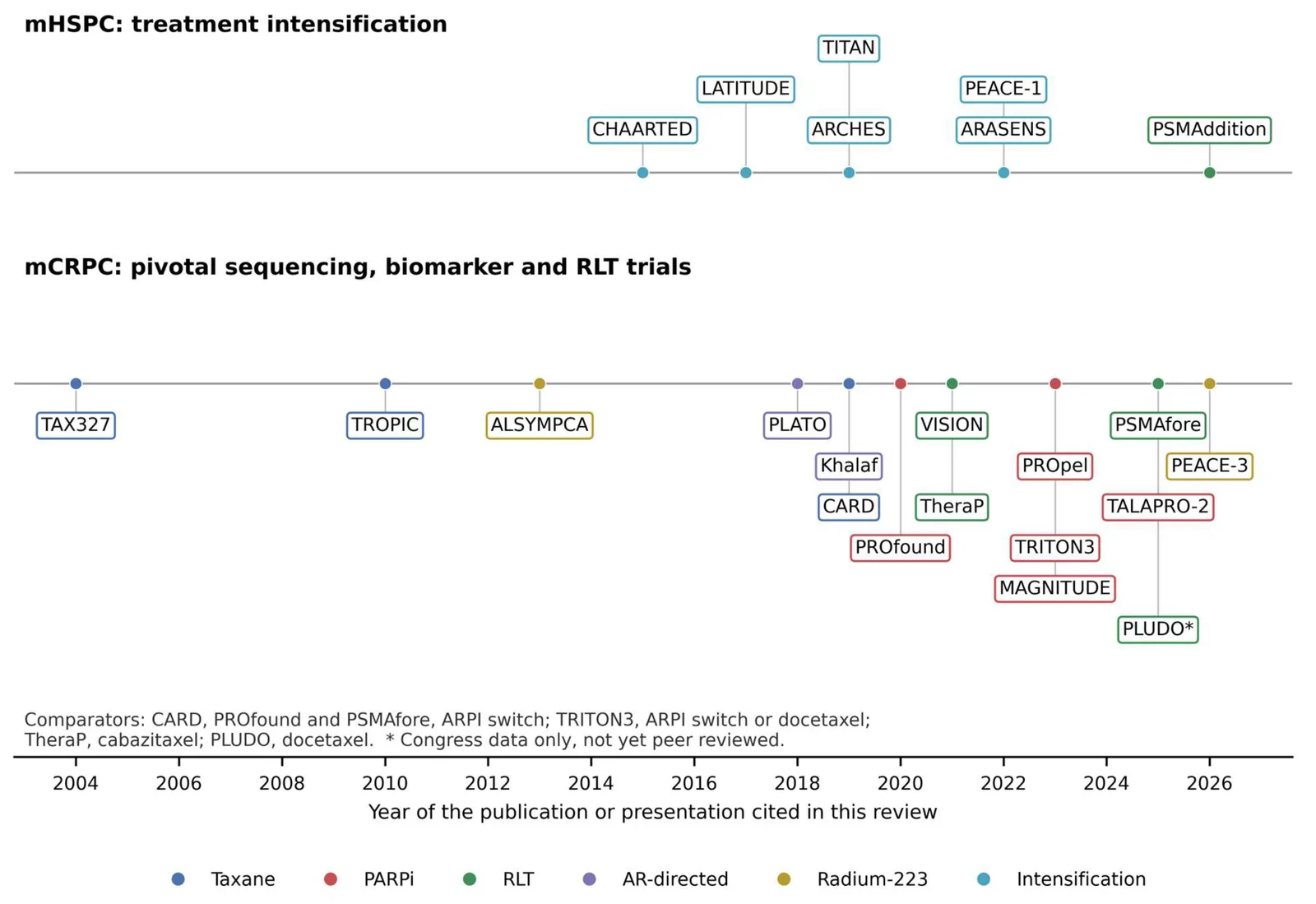
Evolution of the mCRPC treatment landscape and the sequencing evidence gap. Upper row: major treatment-intensification milestones in mHSPC. Lower row: pivotal mCRPC sequencing, biomarker and RLT trials. Trials are plotted by the year of the publication or presentation cited in this review; asterisks denote congress data not yet peer reviewed. The figure emphasises that applicability depends on prior ARPI exposure, taxane exposure, biomarker selection and comparator.

**Figure 2:**
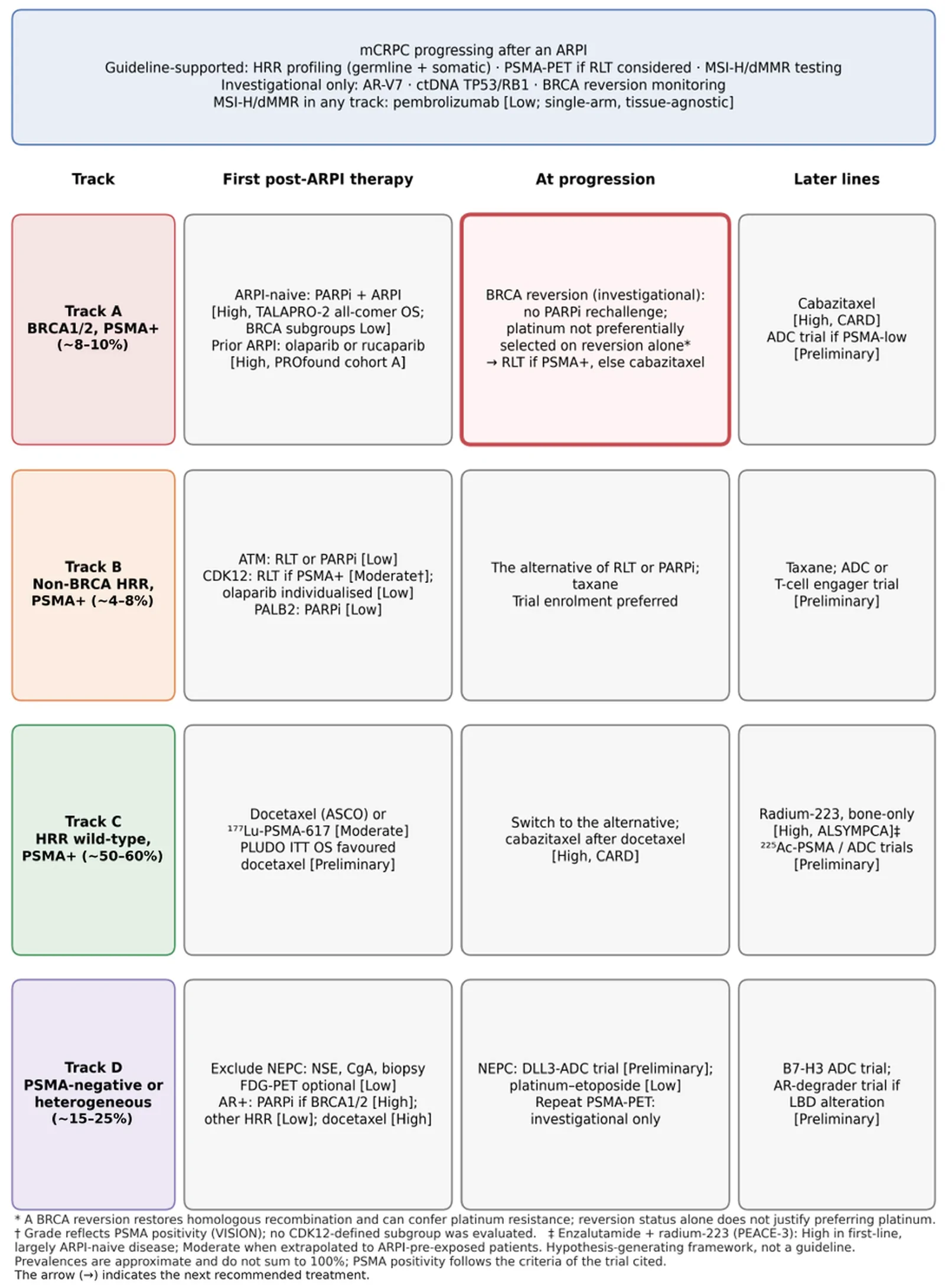
Hypothesis-generating conceptual sequencing framework for mCRPC. Tracks are stratified by HRR alteration status and PSMA-PET expression. The framework is not a guideline and has not been validated by a prospective sequencing trial. PSMA-PET eligibility thresholds differ across trials; clinical judgement, patient preference and trial availability supersede the framework. The arrow (→) indicates the next recommended treatment.

**Figure 3:**
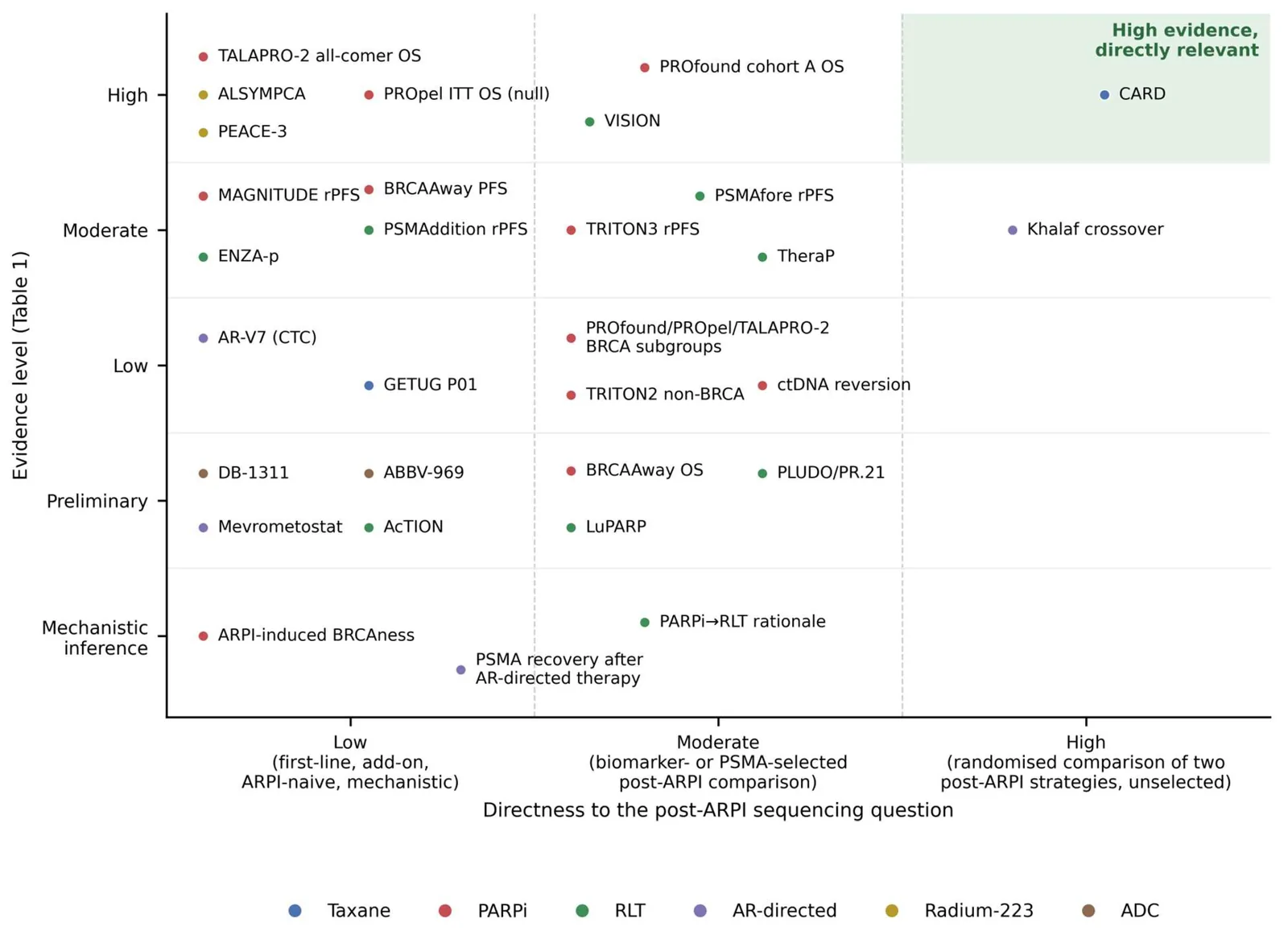
Evidence map of major trials and data sources. The vertical axis shows the evidence level assigned in Table 1, identical to the labels used in the text and Table 2. The horizontal axis shows directness to the post-ARPI sequencing question: high, a randomised comparison of two post-ARPI strategies in a biomarker-unselected population; moderate, a biomarker- or PSMA-selected post-ARPI comparison; low, first-line, add-on, ARPI-naive or mechanistic evidence. Markers are equal-sized and do not encode sample size. PROfound, PROpel and TALAPRO-2 each appear twice: their alpha-controlled OS results (High) and the post hoc BRCA subgroup estimates (Low). BRCAAway is also shown twice: the published PFS result (Moderate) and the congress-only OS update (Preliminary). Only CARD occupies the high-evidence, high-directness zone.

**Table 1:** Evidence hierarchy used throughout this review.

Level	Definition	Examples
High	Phase III trial with a mature, alpha-controlled overall survival endpoint in the population to which the statement applies	VISION OS; CARD OS; PROfound cohort A OS; TALAPRO-2 all-comer OS; PROpel final ITT OS; PEACE-3 final OS; ALSYMPCA
Moderate	Phase III radiographic progression-free survival; randomised phase II with a published, mature endpoint; or phase III OS evidence applied to a population the trial did not directly enrol	PSMAfore rPFS; TheraP; MAGNITUDE rPFS; ENZA-p PSA-PFS; PSMAddition rPFS; BRCAAway PFS; PEACE-3 extrapolated to ARPI-pre-exposed patients; ^177^Lu-PSMA-617 in CDK12-altered disease (VISION)
Low	Single-arm phase I/II, including tissue-agnostic basket studies; retrospective series; post hoc, exploratory or unstratified subgroup analyses of any phase; biomarker association studies without prospective validation	Pembrolizumab in MSI-H/dMMR disease (KEYNOTE-158); PROfound, PROpel and TALAPRO-2 BRCA subgroup analyses; TRITON2 non-BRCA analysis; PROfound gene-level analysis; AR-V7 and ctDNA reversion studies; published ADC phase I/II series
Preliminary	Congress abstract not yet peer reviewed; exploratory analysis presented in abstract form only; first-in-human data	ABBV-969; AcTION; DB-1311; PLUDO; PSMAddition interim OS; BRCAAway OS update; ENZA-p OS; TRITON3 final OS; LuPARP; mevrometostat; SPLASH; ECLIPSE
Mechanistic inference	Biological reasoning without clinical validation	PARPi-to-RLT sequential rationale; ARPI-induced BRCAness; PSMA recovery after AR-directed therapy

**Table 2:** Evidence summary by clinical scenario, with the principal caveat attached to each recommendation.

Setting	Preferred Option	Evidence	Key Data	Major Caveat
BRCA1/2-mutated, ARPI-naive	PARPi + ARPI combination	High for TALAPRO-2 all-comer OS; Low for BRCA subgroup estimates	TALAPRO-2 all-comer OS HR 0.80 [33]; PROpel BRCA OS HR 0.29 [32]; TALAPRO-2 BRCA OS HR 0.50 [33]; BRCAAway OS HR 0.51 vs. olaparib and 0.39 vs. abiraterone [36] (Preliminary)	BRCA estimates are post hoc; limited evidence after prior ARPI exposure in the hormone-sensitive setting
BRCA1/2-mutated, ARPI-pre-exposed	PARPi monotherapy	High (PROfound cohort A OS; Low for the BRCA1/2-specific subgroup estimate)	PROfound cohort A OS HR 0.69 [30]; PROfound BRCA OS HR 0.63 [25]; TRITON3 BRCA OS HR 0.91 [31] (Preliminary)	TRITON3 BRCA OS was not improved; combination benefit unproven after prior ARPI exposure in the hormone-sensitive setting
BRCA1 mutation	PARPi, lower expected magnitude	Low	PROfound BRCA subgroup analysis, BRCA1 *n* = 13 [25]	Very small numbers; do not pool with BRCA2
BRCA reversion at PARPi progression	Avoid PARPi rechallenge; RLT if PSMA-positive, otherwise taxane; platinum not preferentially selected on reversion status alone	Low	ctDNA reversion studies [24,37]	No prospective switching trial; platinum implications remain uncertain
ATM mutation	RLT or PARPi (modest activity)	Low	PROfound and TRITON3 ATM subsets [15,16,31]; mechanism [26]	No head-to-head data; TRITON3 ATM rPFS HR 0.95
CDK12 biallelic loss	RLT if PSMA-positive; trial; individualised consideration of olaparib	Low	TRITON2 non-BRCA analysis [27]; PROfound gene-level analysis [15]; ASCO guideline [28]	Small subgroups; TRITON3 did not enrol CDK12 patients
HRR wild-type, PSMA-positive, taxane-naive	Docetaxel per ASCO guidance, or RLT as an evidence-supported alternative	Moderate	PSMAfore rPFS HR 0.41 [44]; TheraP [47,48]; PLUDO [13,49]; ASCO guideline [28]	No OS gain for RLT versus taxane; PLUDO ITT OS favoured docetaxel; ARPI-switch comparator weak; RLT here diverges from ASCO guidance
Post-ARPI, post-taxane, PSMA-positive	^177^Lu-PSMA-617	High (OS)	VISION OS HR 0.62 [42]	PSMA-PET selection required; thresholds differ across trials
Post-docetaxel and post-ARPI	Cabazitaxel over ARPI switch	High (OS)	CARD OS HR 0.64 [40]	Strongest sequencing dataset in mCRPC, but few if any patients had prior mHSPC ARPI
RLT versus taxane ordering	Order not established; preference-guided	Moderate for TheraP; Preliminary for PLUDO	TheraP [47,48]; PLUDO/PR.21 [13,49]	PLUDO ITT OS favoured docetaxel (HR 1.64, 90% CI 1.14–2.35); crossover analysis is subset-conditioned
RLT versus ARPI switch (rPFS)	RLT is an evidence-supported option	Moderate for rPFS; High for null ITT OS	PSMAfore [44]; SPLASH [45]; ECLIPSE [46]	rPFS benefit but no significant ITT OS difference; 60.3% crossover complicates interpretation
Post-RLT, PSMA-low	ABBV-969 or B7-H3 ADC trial	Preliminary	ABBV-969 ORR 45% among 29 evaluable [14]; DB-1311 ORR 42.3% among 52 evaluable [58]	Phase I; small evaluable population; no prior-RLT-specific efficacy
NEPC/DLL3-positive	DLL3-ADC trial; platinum–etoposide	Preliminary for DLL3-ADC trial; Low for platinum–etoposide	GETUG P01 ORR 8.9% among 46 with measurable disease [65]	No approved NEPC-specific therapy; investigators judged benefit–risk unfavourable
AR-V7 positive	Consider taxane according to prior therapy and clinical eligibility	Low	Antonarakis et al. 2014 [17]	Biomarker association without treatment-selection validation; CTC assay availability limited
AR LBD mutation	AR degrader (rechARge trial)	Preliminary	rechARge phase III [74]	Not yet reported
MSI-H/dMMR	Pembrolizumab in selected patients	Low (single-arm, tissue-agnostic)	Tissue-agnostic MSI-H/dMMR evidence [70]; ASCO guideline [28]	Rare; no prostate-specific randomised trial; KEYNOTE-199 was not a biomarker-selected MSI-H/dMMR study [75]
Bone-predominant, no visceral disease	Radium-223 for symptomatic bone-only disease; enzalutamide + radium-223 in first-line mCRPC	High (ALSYMPCA; PEACE-3 first-line); Moderate for extrapolation to ARPI-pre-exposed patients	ALSYMPCA [71]; PEACE-3 OS HR 0.76 [72]	ALSYMPCA predates ARPI intensification and PEACE-3 was largely ARPI-naive; bone-protecting agent required; avoid combination with abiraterone/prednisone because of ERA-223 [73]

**Table 3:** Pivotal trials referenced in this review, with comparator arm, ARPI-exposure setting and principal limitation.

Trial	Phase	Comparator	ARPI-Exposure Setting	OS HR (95% CI)	rPFS HR	Key Limitation
CARD [40]	III	Abiraterone or enzalutamide (ARPI switch)	Post-ARPI and post-docetaxel; enrolled before ARPI use in mHSPC was standard	0.64 (0.46–0.89)	0.54 (0.40–0.73)	Post-docetaxel only; ARPI-switch comparator
VISION [42]	III	Standard of care (excluding chemotherapy and radium-223)	Post-ARPI and post-taxane	0.62 (0.52–0.74)	0.40	No active taxane control arm
PSMAfore [44]	III	ARPI switch	Post-ARPI, taxane-naive; randomisation stratified by prior-ARPI setting (mHSPC vs. mCRPC)	ITT 0.91 (0.72–1.14), NS; IPCW-adjusted 0.59 (0.38–0.91)	0.41 (primary); 0.49 (updated)	ARPI-switch comparator; 60.3% crossover; IPCW assumes no unmeasured confounding
TheraP [47,48]	II *	Cabazitaxel	Post-docetaxel; prior ARPI permitted	OS similar (RMST 19.1 vs. 19.6 months)	Favoured RLT	Randomised phase II; post-docetaxel only
PLUDO/PR.21 [13,49]	II *	Docetaxel	Post-ARPI, chemotherapy-naive	1.64 (90% CI 1.14–2.35), favouring docetaxel	1.02 (90% CI 0.77–1.35), NS	Congress data only; primary endpoint not met; asymmetric crossover
PROfound [15,25,30]	III	ARPI switch	Post-ARPI	Cohort A 0.69 (0.50–0.97), alpha-controlled; BRCA1/2 0.63 (0.42–0.95), post hoc	0.34 (cohort A); 0.22 (BRCA1/2, post hoc)	67% crossover in cohort A; BRCA analysis post hoc
TRITON3 [16,31]	III	ARPI switch or docetaxel	Post-ARPI, chemotherapy-naive for mCRPC	BRCA1/2 0.91 (0.68–1.20); ATM 1.21 (0.77–1.90) [Preliminary]	0.50 (BRCA1/2); 0.95 (ATM); 0.61 (ITT)	BRCA1/2 or ATM only; no CDK12 cohort; extensive crossover
PROpel [32]	III	Placebo + abiraterone	First-line mCRPC	ITT 0.81 (0.67–1.00), *p* = 0.054, NS; BRCA 0.29 (0.14–0.56), post hoc	0.23 (BRCA, post hoc)	ITT OS not significant; approval restricted to BRCA
TALAPRO-2 [33]	III	Placebo + enzalutamide	First-line mCRPC	All-comer 0.80 (0.66–0.96); HRR-deficient 0.62 (0.48–0.81); BRCA1/2 0.50 (0.32–0.78)	0.47 (HRR-deficient)	All-comer OS is the alpha-controlled result; BRCA subgroup exploratory
MAGNITUDE [34]	III	Placebo + abiraterone	First-line mCRPC	OS benefit not established	0.53 (BRCA)	Futile in non-BRCA HRR and unselected cohorts
BRCAAway [35,36]	II *	Abiraterone or olaparib	First-line mCRPC	vs. olaparib 0.51 (0.22–1.18); vs. abiraterone 0.39 (0.16–0.93) [Preliminary]	PFS vs. olaparib 0.37 (0.17–0.84); vs. abiraterone 0.33 (0.15–0.72)	Small phase II (*n* = 19, 21, 21); OS update is congress data
PSMAddition [12,50]	III	ADT + ARPI alone	mHSPC; ARPI given as part of the regimen in both arms	Interim 0.84, NS, immature [Preliminary]	0.72 (0.58–0.90)	mHSPC setting; OS immature
LuPARP [52]	I	None (single-arm)	Pretreated mCRPC	Not applicable	Not applicable	Single-arm phase I; no randomised comparator
ENZA-p [53,54]	II *	Enzalutamide alone	ARPI-naive by design	0.55 (0.36–0.84) [Preliminary]	PSA-PFS 0.43 (0.29–0.63) (primary endpoint)	ARPI-naive, poor-risk population; 38% crossover
PEACE-3 [72]	III	Enzalutamide alone	First-line mCRPC, largely ARPI-naive	0.76 (0.60–0.96)	0.71 (0.57–0.89)	Bone-protecting agent mandatory from March 2018; more grade ≥3 toxicity with the combination

* Randomised phase II.” The asterisk marks the four randomised phase II trials (TheraP, PLUDO/PR.21, BRCAAway and ENZA-p).

## Data Availability

No new data were created or analysed in this study. All data discussed are available in the cited publications and congress abstracts.

## Abbreviations

Abbreviation	Definition
ADC	antibody–drug conjugate
ADT	androgen-deprivation therapy
ARPI	androgen receptor pathway inhibitor
ctDNA	circulating tumour DNA
dMMR	mismatch repair deficient
FDG	fluorodeoxyglucose
HRR	homologous recombination repair
IPCW	inverse probability of censoring weighting
ITT	intention to treat
mCRPC	metastatic castration-resistant prostate cancer
mHSPC	metastatic hormone-sensitive prostate cancer
MSI-H	microsatellite instability high
NEPC	neuroendocrine prostate cancer
NSE	neuron-specific enolase
ORR	objective response rate
OS	overall survival
PARPi	poly(ADP-ribose) polymerase inhibitor
PSA	prostate-specific antigen
PSMA	prostate-specific membrane antigen
RLT	radioligand therapy
rPFS	radiographic progression-free survival
SUV	standardised uptake value
